# Stability of Reduced and Oxidized Coenzyme Q10 in Finished Products

**DOI:** 10.3390/antiox10030360

**Published:** 2021-02-27

**Authors:** Žane Temova Rakuša, Albin Kristl, Robert Roškar

**Affiliations:** Faculty of Pharmacy, University of Ljubljana, Aškerčeva Cesta 7, 1000 Ljubljana, Slovenia; zane.temova.rakusa@ffa.uni-lj.si (Ž.T.R.); albin.kristl@ffa.uni-lj.si (A.K.)

**Keywords:** assay, coenzyme Q10 content, commercial products, dietary supplements, HPLC-UV, medicines, reducing agent, stability, ubiquinol, ubiquinone

## Abstract

The efficiency of coenzyme Q10 (CoQ10) supplements is closely associated with its content and stability in finished products. This study aimed to provide evidence-based information on the quality and stability of CoQ10 in dietary supplements and medicines. Therefore, ubiquinol, ubiquinone, and total CoQ10 contents were determined by a validated HPLC-UV method in 11 commercial products with defined or undefined CoQ10 form. Both forms were detected in almost all tested products, resulting in a total of CoQ10 content between 82% and 166% of the declared. Ubiquinol, ubiquinone, and total CoQ10 stability in these products were evaluated within three months of accelerated stability testing. Ubiquinol, which is recognized as the less stable form, was properly stabilized. Contrarily, ubiquinone degradation and/or reduction were observed during storage in almost all tested products. These reactions were also detected at ambient temperature within the products’ shelf-lives and confirmed in ubiquinone standard solutions. Ubiquinol, generated by ubiquinone reduction with vitamin C during soft-shell capsules’ storage, may lead to higher bioavailability and health outcomes. However, such conversion and inappropriate content in products, which specify ubiquinone, are unacceptable in terms of regulation. Therefore, proper CoQ10 stabilization through final formulations regardless of the used CoQ10 form is needed.

## 1. Introduction

Coenzyme Q10 (CoQ10) is a lipophilic endogenous compound, with a benzoquinone ring and 10 isoprene side-chain units in its structure [1,2]. Therefore, it is present in two redox forms in the human body: oxidized—ubiquinone (oCoQ10) and reduced form—ubiquinol (rCoQ10). Due to their mutual convention, CoQ10 acts as an excellent electron carrier with a key role in the mitochondrial respiratory chain [3,4]. It is involved in adenosine triphosphate (ATP) production by transferring electrons from complexes I and II to complex III [3,5,6]. In mitochondria, CoQ10 also accepts electrons from enzymes involved in amino acids metabolism (proline dehydrogenase 1, and choline dehydrogenase) [7,8], pyrimidine synthesis (dihydroorotate dehydrogenase) [9], fatty acids oxidation (electron transport flavoprotein dehydrogenase) [10], sulfide detoxification (sulfide quinone oxidoreductase) [11], and mitochondrial glycerol-3-phosphate dehydrogenase, which links oxidative phosphorylation, fatty acid metabolism and glycolysis [12]. Additionally, its reduced form, ubiquinol, is an essential endogenous antioxidant, preventing oxidative lipid, DNA, and protein damage [13,14]. It is also involved in the regeneration of vitamin C and E [15]. Other CoQ10 functions include modulation of gene expression and involvement in inflammation and apoptosis [16,17,18,19].

Coenzyme Q10 is synthesized in the human body, by at least 12 proteins encoded by various genes (COQ genes) [20]. Mutations in any of the COQ genes cause primary CoQ10 deficiency, while secondary deficiency is related to mutations in genes other than COQ or non-genetic causes [20,21]. Secondary CoQ10 deficiency is more common and is associated with aging (significantly reduced synthesis after the age of 30) [22,23,24], nutritional deficiency of CoQ10 or vitamin B6, which is involved in CoQ10 synthesis [16], stress [23], some medical conditions (neurodegenerative disorders, mitochondrial myopathies, cardiomyopathies, fibromyalgia, phenylketonuria, liver cirrhosis) [5,22,25], and use of statins, which inhibit its synthesis [16]. CoQ10 deficiency is associated with reduced ATP production and increased oxidative damage and may lead to various diseases [20]. Five major conditions include encephalomyopathy, severe infantile multisystemic disease, nephropathy, cerebellar ataxia, and isolated myopathy [26]. Patients with CoQ10 deficiency often respond well to CoQ10 supplementation [20,26].

CoQ10 supplements are commonly used not only by patients with CoQ10 deficiency but also by individuals at risk of developing it. Because of its antioxidants’ effects and role in the prevention or treatment of some conditions (atherosclerosis, cardiovascular and neurodegenerative diseases, migraines, infertility, cancer), it is one of the most widely used supplements, along with fish oil and multivitamins [27,28]. CoQ10 supplements are mostly formulated as mono- or multicomponent soft and hard shell capsules and tablets [28].

The two main issues with CoQ10 supplements are low bioavailability and stability. Low oral CoQ10 bioavailability is a result of its high molecular weight (863 g/mol), lipophilicity, and water insolubility, leading to slow and incomplete absorption in the gastrointestinal tract [28,29,30,31]. With modern pharmaceutical development, several approaches, such as self-emulsifying systems, nanosystems, cyclodextrin complexation, and solid dispersions, have been undertaken to maximize its bioavailability [32,33,34]. In this manner, one of the most prominent advances is the pharmaceutical formulation of CoQ10 in its reduced form. Its bioavailability has been found up to 4.8 fold greater in comparison with oCoQ10, as reported by an increasing number of studies [23,24,29,30,35,36,37,38,39]. A part of these studies focuses on the elderly, for which CoQ10 supplementation is most relevant [29,35]. However, several studies highlight the importance of the formulation (solubilization, vehicle composition, presence of antioxidants) and high inter- and intraindividual variations on CoQ10 bioavailability rather than the administered CoQ10 form [31,40,41,42]. Nonetheless, Langsjoen et al. [24] reported higher rCoQ10 than oCoQ10 bioavailability by using identical soft gel capsule excipients. An additional advantage of rCoQ10 supplementation is its genuine antioxidant activity, while oCoQ10 requires enzymatic reduction before acting as an antioxidant [43]. The enzymatic activities of coenzyme Q reductases change according to physiological and pathological conditions and decrease with age, use of some medications, and after intense physical activities [44,45,46]. Supplementation with rCoQ10, therefore, seems advantageous over oCoQ10, especially in the above-mentioned cases. Consequently, an increase in the number of commercially available rCoQ10 preparations has been evident in the past few years.

The stability issues associated with CoQ10 are more pronounced with the use of rCoQ10, which is quickly oxidized, when in contact with air or after light exposure [44,47,48]. Providing quality rCoQ10 products is therefore quite demanding in terms of obtaining a stable formulation [28,47]. However, the stability of oCoQ10 is also affected by some environmental conditions (UV-light and temperature [48,49,50,51,52]) as well as the presence of reducents in the products [52].

Even though the general CoQ10 instability is described in the literature, its stability as well as the stability of its individual forms in finished products, which are used by numerous consumers, has not yet been evaluated. Since the health outcomes are dependent on the applied CoQ10 form and its stability, this study aimed to evaluate the content and stability of rCoQ10 and oCoQ10 in commercial supplements and medicines in view of their quality and consequently efficacy.

## 2. Materials and Methods

### 2.1. Chemicals and Reagents

Ubiquinone (oCoQ10) (≥98%), ubiquinol (rCoQ10) (≥97%), and DL-*α*-tocopherol (≥97%) were purchased from Carbosynth (Berkshire, England). Anhydrous iron(II) chloride (≥98%) and iron(III) chloride (≥98%), ascorbic acid (≥99%), β-carotene (≥97%), 1,4-dithiothreitol (DTT) (≥99%), ellagic acid (≥95%), sodium sulfide (≥99%), quercetin (≥98%), 85% orthophosphoric acid (H_3_PO_4_), and HPLC grade solvents: acetonitrile (ACN), methanol (MeOH), *n*-hexane and tetrahydrofuran (THF) were obtained from Sigma-Aldrich (Steinheim, Germany). Anhydrous ethanol (EtOH) was obtained from Merck (Darmstadt, Germany). Ultra-pure water was obtained through a Milli-Q water purification system A10 Advantage (Millipore Corporation, Bedford, MA, USA).

### 2.2. High-Performance Liquid Chromatography (HPLC) Analysis

The contents of oCoQ10, rCoQ10, and total CoQ10 in the tested preparations at each time point were measured by an HPLC-UV method, which had previously been validated for the quantification of both CoQ10 forms in finished products [53]. The analysis was performed on an Agilent 1100/1200 Series instrument (Agilent Technologies, Santa Clara, CA, USA) equipped with a UV-VIS detector and ChemStation data acquisition system. The chromatographic separation was performed on a reversed-phase Luna C18 (2) 150 × 4.6 mm, 3 μm particle size column (Phenomenex, Torrance, CA, USA) at 25 °C. The mobile phase consisted of ACN:THF:Milli-Q water (50:45:5, *v*/*v*/*v*) was utilized in isocratic elution mode at a flow rate of 1 mL/min. The detection wavelength was 280 nm and the injection volume was 20 µL.

### 2.3. Samples

Eleven products, ten registered as dietary supplements (DS) and one as a non-prescription medicine (NPM) were purchased in Slovenia and analyzed for oCoQ10, rCoQ10, and total CoQ10 content. These included: BioActive Q10 Uniqinol^®^ 30 mg with vitamin C, Pharma Nord (Denmark); Bio-Qinon^®^ Active Q10 30 mg with vitamin C, Pharma Nord (Denmark); CoQ10, Now (USA); Fidi koencim 10^®^, Fidimed, PharmaSwiss (Slovenia); Imuno BC plus, Yasenka (Croatia); Koencim Q10, Lekarna Ljubljana (Slovenia); Koencim Q10^DE^, Marifit (Germany); MorEPA Gold Smart Fats^®^, Minami (Netherlands); Omega 3 complex, Dr. Böhm^®^ (Austria); Supradyn energija Q10, Bayer (France); and Ubiquinol CoQH-CFTM, Now (USA). More detailed data are summarized in Table 1.

### 2.4. Sample Preparation

The sample preparation procedure was based on a published procedure for the simultaneous content evaluation of the individual CoQ10 forms (oCoQ10 and rCoQ10) [53]. Soft-shell capsules were initially cut in half and hard-shell capsules were opened. The content, as well as the shell of each capsule, were quantitatively transferred to a 100 mL volumetric flask, which was filled to approximately ¾ of its volume with *n*-hexane. The samples were further sonicated for 10 min and then shaken for 20 min with a rotary mixer. After filling the flask to the mark with the same solvent, the samples were manually shaken and centrifuged for 10 min at 2640× *g* and 25 °C. A part of the supernatant (500 μL) was evaporated to dryness for approximately 15 min under a stream of nitrogen at 40 °C. The dry residues were reconstituted with ACN (1 mL), sonicated for 2 min, mixed with a vortex for 30 s, transferred into a vial, and immediately analyzed. To confirm total CoQ10 content, dry residues of each sample were additionally reconstituted with 0.01% Fe^3+^ solution in EtOH (1 mL), followed by the procedure described above (sonication, vortex mixing, and analysis).

The initial part of the sample preparation procedure for the tested tablets was as follows: one tablet was firstly powdered and added to 0.1% H_3_PO_4_ solution (2.0 mL), followed by vortex mixing (10 min). Furthermore, 8 mL of *n*-hexane were added, followed by 10 min of sonication and 2 min of vortex mixing. The samples were centrifuged (2640× *g*, 25 °C, 10 min) and proceeded as described above.

### 2.5. Stability Study

#### 2.5.1. Accelerated Stability Study

Accelerated stability testing was performed on all products (Section 2.3) by the ICH Q1A (R2) guidelines. The tested products in their original packaging were exposed to elevated temperature (40 °C) and relative humidity (75% RH) in a climate chamber (ICH 260 L, Memmert, Büchenbach, Germany). The contents of rCoQ10, oCoQ10, and total CoQ10 were evaluated immediately after opening as well as after 2 weeks, 1, 2, and 3 months by a published procedure for rCoQ10, oCoQ10, and total CoQ10 quantification [53]. All samples were prepared in at least triplicate. During the stability study, all products were within their expiration dates. 

#### 2.5.2. Long-Term (Real-Time) Stability Study

The long-term stability was evaluated on one of the tested products (product 11 in Table 1). Real-time stability was assessed by determining the contents of rCoQ10, oCoQ10, and total CoQ10 in five different LOTs of the same product—2, 6, 10, 18, and 30 months after production months by the published procedure for rCoQ10, oCoQ10, and total CoQ10 quantification [53]. The products had been stored in their original packaging at room temperature before analysis and were within their expiration date except for the LOT analyzed 30 months after production. 

#### 2.5.3. Stability of Standard oCoQ10 Solutions in the Presence of Antioxidants

The stability of oCoQ10 was evaluated in simple solutions containing only oCoQ10 or also some antioxidants and reducing agents. The concentration of oCoQ10 was 29 μM. The antioxidants were added in the following concentrations: 2.8 mM FeCl_2_, 1.9 mM ascorbic acid, DTT, and Na_2_S, 1.0 mM for ellagic acid and quercetin, 0.5 mM for DL-*α*-tocopherol, and 50 μM for β-carotene. In addition to these high concentrations, 10-fold lower (medium) and 100-fold lower (low) concentrations were tested. All solutions were prepared in triplicate, and in anhydrous ethanol as solvent. The stability of oCoQ10 was evaluated for nine days of storage at 40 °C at regular time points.

#### 2.5.4. Degradation Kinetics

Zero, first, and second-order kinetics were fitted to the data obtained within accelerated and long-term stability studies as well as in standard solutions to evaluate the kinetics of CoQ10 degradation. The most suitable kinetic order was selected according to the correlation coefficient and applied to determine the degradation rate constant (k). The temperature effect on the degradation kinetics of CoQ10 was determined using the Van’t Hoff equation. The calculated degradation constants at ambient temperature were used for shelf-life determination, which was defined as the time necessary for CoQ10 degradation to 90% of its declared content (t_90%_).

## 3. Results

### 3.1. Accelerated Stability Study

Accelerated stability study was performed on eleven tested products with CoQ10, including commercial dietary supplements and non-prescription medicine, which were stored in their original containers at 40 °C and 75% RH. Based on the results, rCoQ10 and oCoQ10 contents were calculated at each time point (immediately after opening and after 2 weeks, 1, 2, and 3 months). The contents of rCoQ10 and oCoQ10 are presented as a percentage of the CoQ10 content declared on the label of the products. The results are grouped into three categories according to the labeled CoQ10 form. 

#### 3.1.1. Products with Undefined CoQ10 Form

**Figure 1:**
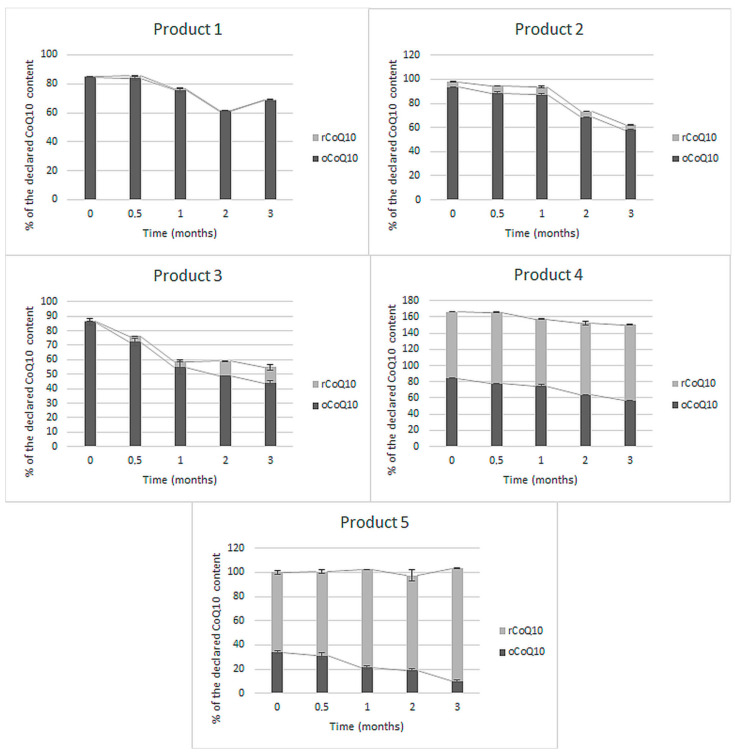
The stability of rCoQ10, oCoQ10, and total CoQ10 in the five tested finished products with undefined CoQ10 form after the accelerated stability study.

Five of the eleven tested finished products did not specify the contained CoQ10 form on their label nor in the ingredients lists. The obtained results, representing the initial rCoQ10, oCoQ10, and total CoQ10 change during three months of accelerated stability study, are presented in Figure 1.

As can be seen in Figure 1, total CoQ10 contents in the five tested finished products, which did not specify CoQ10 form ranged between 82% and 166% of the label claim. The first three products (products 1, 2, and 3) contained predominantly oCoQ10. Evaluating their stability, a similar pattern may be observed—a decline in oCoQ10 content by 16% to 42% of the initial oCoQ10 content, which was not (products 1 and 2) or not solely (product 3) a consequence of its reduction to rCoQ10. Among these three products, oCoQ10 conversion to rCoQ10 was highest in product 3, reaching about 20% of total CoQ10 by the end of the study. A slightly higher reduction extent was observed in product 4, which initially contained approximately the same amounts of rCoQ10 and oCoQ10. A significantly higher, almost total reduction was observed in product 5, which initially contained a larger share of rCoQ10. Total CoQ10 content after three months of storage at elevated temperature and RH remained approximately the same in product 5 and declined by about 15% in products 1 and 4 and by more than 30% in products 2 and 3.

#### 3.1.2. Products with CoQ10 in the Form of Ubiquinol

**Figure 2:**
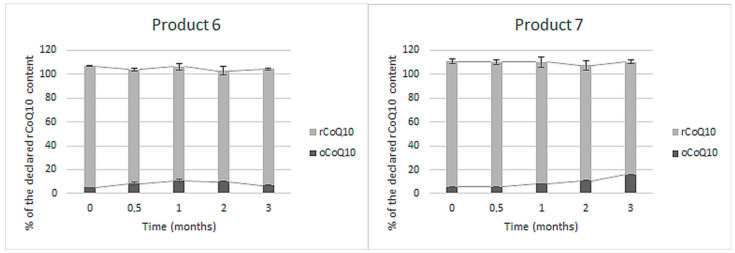
The stability of rCoQ10, oCoQ10, and total CoQ10 in the two tested finished products with specified rCoQ10 content after the accelerated stability study.

The content of rCoQ10 was specified in two of the eleven tested finished products. The stability of rCoQ10 and its conversion into oCoQ10 in these two products within the accelerated stability study is presented in Figure 2. 

Both products initially contained about the same rCoQ10 content as declared (102–105%), and additional oCoQ10, which accounted for approximately 5% of the declared rCoQ10 content. A lesser share of rCoQ10 (up to 10%) was additionally converted into oCoQ10 within the accelerated stability study. Total CoQ10 content did not significantly change in any of the two products by the end of the study.

#### 3.1.3. Products with CoQ10 in the Form of Ubiquinone

**Figure 3:**
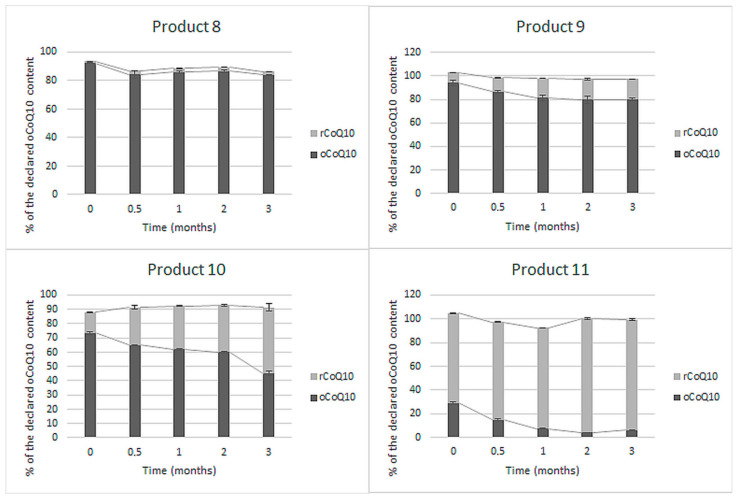
The stability of rCoQ10, oCoQ10, and total CoQ10 in the four tested finished products with specified oCoQ10 content after the accelerated stability study.

Four of the eleven tested finished products specified oCoQ10 content on their label. In addition to the labeled oCoQ10, the share of rCoQ10 contributing to total CoQ10 content was determined at each time point during the three months of accelerated stability study (Figure 3).

The determined oCoQ10 contents in these products were between 29% and 94% of the label claims. Total CoQ10 content was near the labeled oCoQ10 content (88–105%). The initial rCoQ10 share was between 1 and 75% of the declared CoQ10 content. Product 8 initially contained 92% of the labeled oCoQ10 amount and only minimal amounts of rCoQ10. A slight decline in oCoQ10 and total CoQ10 content and insignificant oCoQ10 to rCoQ10 conversion were observed by the end of the accelerated stability study. Similarly, total CoQ10 did not significantly change during storage of products 9 and 10. However, a larger extent (about 10% in product 9 and almost 40% in product 10) of the initial oCoQ10 converted into rCoQ10 by the end of the study. The biggest discrepancy between labeled and contained oCoQ10 amount was observed in product 11, which is registered as non-prescription medicine. This product initially contained less than 30% of the labeled oCoQ10 content, despite the high total CoQ10 content (104%). The initially present oCoQ10 almost completely converted into rCoQ10 by the end of the study, while total CoQ10 remained almost the same.

### 3.2. Long-Term (Real-Time) Stability Study

**Figure 4:**
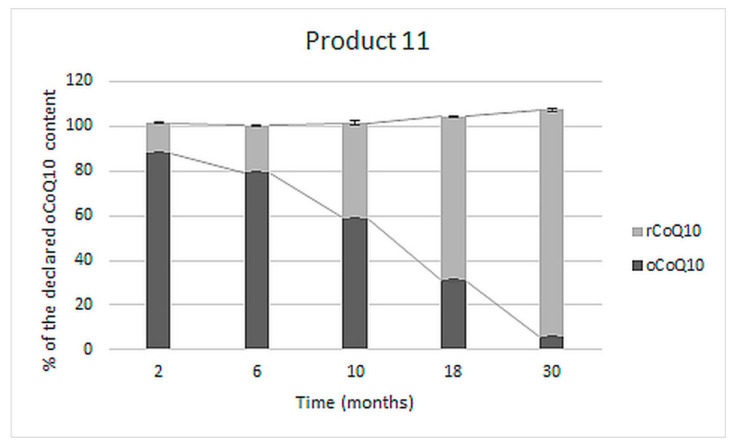
Long-term stability of rCoQ10, oCoQ10, and total CoQ10 in a finished product with specified oCoQ10 content evaluated at different time points after production.

A long-term stability study was performed on one of the tested products (product 11), which showed the most significant deviations from the label claims during the accelerated stability study. The effect of storage at room temperature on the reduction of oCoQ10 was evaluated by determining the contents of rCoQ10, oCoQ10, and total CoQ10 in five different LOTs of the product stored unopened in their original packaging for 2, 6, 10, 18, and 30 months after production. The obtained results are presented in Figure 4. 

As evident, shortly after production (two months), the majority (almost 90%) of the determined CoQ10 was in its oxidized form, which was gradually reduced to rCoQ10 during storage. In the LOT analyzed after its defined two-year shelf-life (30 months after production), an almost negligible part of total CoQ10 content was in its declared, oxidized form (about 5%). The total CoQ10 content did not significantly change and was comparable in all five tested LOTs of the product.

### 3.3. CoQ10 Degradation Kinetics in the Tested Products

Within the accelerated stability study, oCoQ10 was found more unstable than rCoQ10, as its conversion to rQ10 and other degradation processes occurred in the majority of the tested products. Therefore, these reactions were kinetically evaluated and contributed to the reaction rate constants. The decline of oCoQ10 in the tested products followed the first-order kinetics, which was used for the calculation of degradation rate constants and shelf-lives prediction under the tested accelerated conditions (Table 2). Additionally, the rate constant at ambient temperature (20 °C) was calculated for product 11, which was exposed to long-term stability testing. After obtaining the degradation rate constants at different temperatures, we applied a simple approach based on the Van’t Hoff equation for the prediction of stability and shelf-lives at ambient temperature. Initially, the reaction quotient Q was calculated, which was 3.70. Assuming the same temperature relation, degradation rate constants, and shelf-lives at ambient temperature were also calculated for all other products (Table 2).

The decline in oCoQ10 contents as a result of its degradation and conversion to rCoQ10 were formulation dependent with rate constants, which differ by up to 50-fold (Table 2). Van’t Hoff temperature relation was applied to calculate the products’ shelf-lives at ambient temperature. The predicted shelf-lives ranged between 1.1 and 11.0 months with an average of 6.7 months for all products, except for product 8, which had a significantly longer shelf-life. The predicted shelf-lives are most meaningful for products with specified oCoQ10 content (products 8–11 in Table 2). 

The highest degradation constants were observed in products 11, 5, 3, and 2, which contained lower oCoQ10 amounts (≥10 mg per capsule), which implied that its stability may be concentration-dependent. In addition, products 11 and 5 contained lower shares of total CoQ10 in the oxidized form. More than half of the initially present oCoQ10 in these two products had converted into rCoQ10 before the initial analysis, which was performed within the products’ shelf-lives. 

Moreover, we evaluated the impact of formulation, more specifically the declared antioxidants and their contents, on oCoQ10 stability. As may be seen in Table 1 and Table 2, products 8, 4, and 9, which had the lowest rate constants, contained only vitamin E. On the other hand, products 5, 3, and 2 with high oCoQ10 rate constants also had high vitamin C contents (between 80 and 90 mg per capsule). Product 5 additionally contained rosemary extract, which also has reducing and antioxidant activity [47]. In the case of product 11, which had the highest oCoQ10 rate constant, vitamin C was declared, but its content was not specified. The carrier oil is also a noteworthy factor to consider when studying oCoQ10 stability within formulations. Since the manufacturers rarely specify the oil content (Table 1), a quantitative relation could not be established. The obtained results (Table 2) imply that the oil type is more important for oCoQ10 stability than the presence of different oils. Namely, product 11, with the highest oCoQ10 rate constant contained only corn oil, while product 8, with the lowest rate constant, contained a combination of sunflower, coconut, and palm oil. In addition, CoQ10 stability may be affected by the presence of impurities within the formulation, and especially in the CoQ10 raw materials, which differ among the manufactures. However, the purity and the presence of impurities in the used CoQ10 raw material are generally not specified by the manufacturers and were unknown for the tested products.

### 3.4. Stability of oCoQ10 Standard in the Presence of Antioxidants 

The stability of oCoQ10 and its conversion to rCoQ10 was also evaluated in standard solutions in the presence of antioxidants and reducing agents. The observed conversion of oCoQ10 to rCoQ10 was kinetically evaluated by first-order reaction rate, which was applied for the calculation of rate constants (k). The obtained results are presented in Table 3.

It is evident that oCoQ10 is stable in its dissolved form and also remained unaltered in the presence of ellagic acid, vitamin E, quercetin, iron(II) chloride, and β-carotene, irrespective of their concentration. On the other hand, DTT, Na_2_S, and vitamin C were quite effective in the reduction of oCoQ10. Their reducing capacity was concentration dependent.

## 4. Discussion

CoQ10 supplements are commonly used worldwide [54] for the prevention and treatment of cardiovascular [55,56,57], metabolic [58,59,60], neurodegenerative [61], kidney and liver diseases [8], migraines [62], glaucoma [63], some types of cancer [64], statin-induced adverse effects [65,66], infertility [67], and other conditions. However, the formulation and use of quality, safe, and efficient CoQ10 supplements and medicines is challenging because of solubility and thus bioavailability and also stability concerns [28,68]. While extensive research has been recently focused on the improvement of its bioavailability, less attention is paid to the stability issues. In general, the stability of CoQ10 is affected by heat, light, and oxygen [28]. Some of the researched approaches to enhance CoQ10 bioavailability, such as complexation with γ-cyclodextrins [69], use of self-emulsifying delivery systems [70], or solid dispersions [71] may also lead to increased CoQ10 stability. However, the CoQ10 stability issues were not addressed within these studies. The use of liposomal and microencapsulated CoQ10 are promising approaches towards CoQ10 bioavailability and stability improvement, which require further research before their use in clinical practice [72,73,74,75]. 

Currently, CoQ10 is mostly dispersed in oil in soft-shell capsules or incorporated as a crystalline powder in hard-shell capsules and tablets [28]. Contemporary CoQ10 products on the market declare the content of an individual form—rCoQ10 or oCoQ10. Within this study, we evaluated the content and stability of oCoQ10 and rCoQ10 in eleven dietary supplements and medicines from the Slovenian market, including five products with undefined CoQ10 form, and six products with specified CoQ10 form. For this purpose, we used a validated HPLC-UV method, which is advantageous over the current regulatory methods in terms of the chromatographic separation and simultaneous quantification of both CoQ10 forms. In comparison, the simultaneous rCoQ10 and oCoQ10 determination is not feasible due to improper selectivity of the spectroscopic method proposed by the Eur. Ph. [76] and due to the sample preparation procedure, based on the conversion of all present CoQ10 into oCoQ10, proposed by the USP [77,78] and AOAC [79]). Additional data on the adequacy of the applied method for quantification of both rCoQ10 and oCoQ10 from a technical point of view are provided in the Supplementary Materials (Tables S1–S3). Within the interpretation of the results on CoQ10 contents, the acceptable tolerances in dietary supplements, provided by the USP, were considered. As stated in the monographs Ubidecarenone Tablets and Ubidecarenone Capsules, both should contain not less than 90% and not more than 115% of the labeled oCoQ10 content [72,73]. The same acceptable tolerances are provided for rCoQ10 in Ubiquinol Capsules [74] and were also applied for total CoQ10 content on products with undefined CoQ10 form. Summarily, we determined initial CoQ10 contents between 29% and 166% of the labeled claims. Five of the eleven tested products were outside the acceptable range (Figure 1, Figure 2 and Figure 3), mostly due to lower contents than declared. One of them (product 4 in Figure 1) contained significantly higher total CoQ10 content than labeled (above the acceptable tolerance), which was probably added to achieve better clinical outcomes and not to compensate for CoQ10 losses during the storage of the product. An additional conclusion, which can be drawn from these results, is that almost all tested CoQ10 products contained at least minimal amounts of both its forms (Figure 1, Figure 2 and Figure 3). The co-occurrence of rCoQ10 in products, which specify oCoQ10 contents, has also been reported in previous studies. About 40% of the 62 tested oCoQ10 supplements from the Japanese market contained rCoQ10 in amounts between 4% and 88% of total CoQ10 content. rCoQ10 was also determined in half of the 12 tested oCoQ10 supplements from the Hungarian market, which accounted for up to 16% of total CoQ10 content [80]. However, the results of these studies on rCoQ10 and oCoQ10 contents in the absence of stability studies are inconclusive whether the determined rCoQ10 had been intentionally added by the manufacturers to provide greater bioavailability and efficiency or it originates from oCoQ10 reduction during storage [80]. Namely, Kettawan et al. demonstrated that rCoQ10 can be produced by the interaction of oCoQ10 with vitamins E (*α*-tocopherol) and/or vitamin C (ascorbic acid) during storage of experimental formulas in the form of soft-shell capsules and liquid products, but not in hard-shell capsules [81]. However, the stability of CoQ10 in finished products, including its individual forms and total CoQ10, has not yet been evaluated. 

By performing accelerated stability study on a variety of products with different contents of the individual CoQ10 forms, we aimed to provide evidence-based information on CoQ10 stability in finished products and thus also on the truthfulness of the label claims and quality of the products, found on our market. Thereby, we evaluated the changes in oCoQ10 and rCoQ10 contents, excluding other degradation products. Evaluating CoQ10 stability under accelerated storage conditions in finished products, with undefined CoQ10 form, all five tested products were found dynamic, as significant oCoQ10 reduction and total degradation were observed (Figure 1). oCoQ10 reduction predominantly occurred in soft-shell capsules, whereas its degradation was more characteristic in hard-shell capsules. We can, therefore, conclude that the carrier significantly affects the course of oCoQ10 reactions, along with the presence of excipients, which is also supported by the findings by Kettawan et al. [81]. These reactions had a diverse effect on total CoQ10 content at the end of the study, resulting in a significant decline (by almost 40%) in four of these five products and unchanged content in one product. Despite such declines in oCoQ10 content, we did not detect any considerable chromatographic peaks under the used chromatographic conditions (chromatograms in Supplementary Materials (Tables S1–S3)). Further research is needed for the clarification of CoQ10 degradation mechanisms and identification of its degradation products, which is a topic of current interest [82,83]. These obtained results lead to the conclusion that, although oCoQ10 is considered as the more stable CoQ10 form, its instability within formulations should not be neglected. 

Further on, we evaluated CoQ10 stability under accelerated storage conditions in six products, which declare the content of an individual CoQ10 form. In these products, the declared form in the formulation should be stable in its original form, meaning that it is not converted to another form or species and its content is not significantly diminished during storage [84]. Two of the eleven tested products were labeled to contain only rCoQ10, which is promoted as the superior CoQ10 form regarding the bioavailability, however more demanding in terms of stability [68,85]. Because of its susceptibility to oxidation, rCoQ10 requires proper stabilization through its final formulations [85]. An interesting finding from our study is that both tested rCoQ10 products had appropriate initial rCoQ10 contents and provided acceptable stability, which was exemplary in the case of product 6 (Figure 2). Even though some rCoQ10 oxidation occurred during storage of product 7, the manufacturer provided adequate rCoQ10 content by the addition of excess rCoQ10 amounts (overage). Manufacturers frequently apply this principle when formulating unstable compounds (such as vitamins) to ensure proper contents throughout the shelf-life and compensate losses during production or storage [86,87,88]. 

Within the accelerated stability study on products with declared oCoQ10 content, all in the form of soft-shell capsules, oCoQ10 degradation, and especially conversion to rCoQ10 was observed (Figure 3). The initially determined oCoQ10 were below the USP acceptable ranges in half of the tested products, and below these limits in all four tested products by the end of the study. Such inadequate oCoQ10 contents and the presence of non-listed rCoQ10 are inappropriate from a regulatory aspect. Similarly, as noted within the accelerated stability study of products with undefined CoQ10 form, the conversion of oCoQ10 to rCoQ10 was insignificant in products, which contained minimal amounts of rCoQ10 at the beginning of the study (product 8 in Figure 3). Initial rCoQ10 content and reduction extents were higher in the remaining three products. Inadequate oCoQ10 content was most pronounced in the tested non-prescription medicine (product 11 in Figure 3). The most probable reason for inappropriate oCoQ10 content is its instability within this formulation in the presence of various oxido-reductive agents (e.g., vitamin C) since further, almost complete reduction was observed within the accelerated stability study. This hypothesis was proven by a long-term (real-time) stability study performed on five LOTs of this product, analyzed at different time points after production. The results, presented in Figure 4, reveal that oCoQ10 reduction, observed within the accelerated stability study (Figure 3) also occurred during storage at ambient temperature. Furthermore, the conversion occurred to a great degree, leading to about a 50% reduction of CoQ10 within the first year of storage and almost total reduction after the second year (shelf-life), while total CoQ10 remained almost unchanged. Although the presence of rCoQ10 may be beneficial from a clinical point of view, it is inappropriate from the regulatory aspect, particularly in non-prescription medicines.

Degradation kinetic evaluation of the observed reactions demonstrated that oCoQ10 stability depended on the formulation itself (Table 2). The instability of oCoQ10, leading to reduction or other degradation processes within formulations, may be associated with its lower contents than declared. However, further research on a wider range of CoQ10 products would be needed to establish this relationship. Summarizing the results in Table 2, we can conclude that CoQ10 degradation was more pronounced in the three tested products (products 1–3) in the form of hard-shell capsules or tablets (total CoQ10 between 60 and 80% of the initial), whereas total CoQ10 in the tested products in the form of soft-shell capsules remained within 10% of the initial content by the end of the accelerated stability study. In these products, oCoQ10 reduction was more typical. Therefore, adequate mobility of oCoQ10 within formulations, generally provided by the oil content in soft-shell capsules, is a prerequisite for its reduction.

Providing that this condition is fulfilled, we also noticed a correlation between higher reaction rate constants and lower initial share of oCoQ10 in relation to total CoQ10. This implicates that some of the finished products had been formulated as oCoQ10 products; however, due to improper stabilization, they provide conditions for oCoQ10 reduction during their usage and storage. In these terms, vitamin C was identified as the key reducing agent for oCoQ10 conversion to rCoQ10 in commercial products in the form of soft-shell capsules. Correlation between oCoQ10 reduction and the labeled presence of ascorbic acid in the tested products, especially in higher amounts, was observed, while the presence of different vitamin E amounts did not seem to cause oCoQ10 reduction. 

For further clarification of the observed oCoQ10 reduction in the tested products, we additionally evaluated its stability in simpler systems—oCoQ10 standard solutions with and without added antioxidants. In addition, their concentrations were selected to obtain comparable CoQ10—antioxidant molar ratios as in the finished products. Based on the results (Table 3), it can be concluded that potent reducing agents, such as vitamin C, DTT, or sodium sulfide, are required for oCoQ10 reduction. The observed correlation between their concentration and rate constants (Table 2) supports our findings within the accelerated stability study, that oCoQ10 reduction is enhanced in the presence of higher amounts of reducents (vitamin C) within the formulation. Since oCoQ10 reduction in solutions containing tocopherols (DL-α-tocopherol), flavonoids (quercetin), polyphenols (ellagic acid), carotenoids (β-carotene), and other reducing agents (FeCl_2_) did not occur, these results imply that they would less likely cause its reduction within formulations. 

The findings from this study emphasize the importance of proper CoQ10 stabilization through final formulations, regardless of the used CoQ10 form. Appropriate CoQ10 contents throughout the shelf-lives of the products, which are typically two years or longer, are required to achieve the desired health effects. This study also provides certain explanations for the inappropriate CoQ10 contents in supplements, reported in Japan [81], Germany [89], Hungary [80], and Slovenia [53,90]. 

## 5. Conclusions

The efficiency and consequently health outcomes of CoQ10 supplements are directly linked to its stability in the finished products. Contemporary CoQ10 supplements contain only one of the two CoQ10 redox forms—rCoQ10 or oCoQ10. The individual forms differ in their stability, which is also related to the presence of excipients in the finished products. The findings of this study suggest that manufacturers pay more attention to the stabilization of rCoQ10, which is easily oxidized to oCoQ10, than to oCoQ10. Degradation of oCoQ10 as well as its conversion to rCoQ10 were typically observed within our accelerated stability study, performed on eleven CoQ10 commercial dietary supplements and medicines on the Slovenian market. The presence of vitamin C in a suitable medium was identified as the key element for oCoQ10 reduction during the products’ storage. The conducted long-term (real-time) stability study revealed that these conversions also occur at ambient temperature and within the shelf-life of the products. Although beneficial from a clinical point of view, oCoQ10 reduction and consequently decline in its content in products, with specified oCoQ10 content, is inappropriate from the regulatory aspect. The observed conversion of oCoQ10 to rCoQ10 during storage of the products may also be associated with different and conflicting information on their bioavailability within clinical studies.

## Figures and Tables

**Table 1:** Data on the tested products.

Product	Type	Form	Declared CoQ10		Declared Antioxidants	Declared Carrier Oil
			Form	Content (mg)		
1	DS	h. cap.	CoQ10	30	40 mg vit C, 16 mg vit E ^a^	/
2	DS	tbl.	CoQ10	4.5	80 mg vit C, 12 mg vit E ^a^, 800 µg vit A	/
3	DS	h. cap.	CoQ10	10	90 mg vit C	/
4	DS	s. cap.	CoQ10	30	12 mg vit E ^a^	cold water fish oil (1150 mg)
5	DS	s. cap.	CoQ10	30	80 mg vit C, rosemary extract, and tocopherols	deep-sea fish oil concentrate (787 mg), olive oil, and garlic etheric oil extract
6	DS	s. cap.	rCoQ10	30	12 mg vit C, mixture of tocopherols	coconut oil
7	DS	s. cap.	rCoQ10	50	capric, caprylic, and α-lipoic acid	/
8	DS	s. cap.	oCoQ10	50	6 mg vit E ^b^	sunflower, coconut, and palm oil
9	DS	s. cap.	oCoQ10	50	20 mg vit E ^a^	rice bran oil
10	DS	s. cap.	oCoQ10	30	25 mg vit C	palm oil
11	NPM	s. cap.	oCoQ10	30	24 mg vit E ^a^, vit C, β-carotene	soybean oilcorn oil

DS—dietary supplements; NPM—non-prescription medicine; s. cap.—soft-shell capsules; h. cap.—hard-shell capsules; tbl.—tablets; vit C—vitamin C; vit E—vitamin E; ^a^ in the form of DL-α –tocopheryl acetate; ^b^ in the form of DL-α –tocopherol.

**Table 2:** Degradation of oCoQ10 in the tested products and extrapolation to ambient temperature.

Product	Initial oCoQ10 Content (mg ± SE)	Initial oCoQ10 Share of Total CoQ10 (%)	40 °C				20 °C
			Remaining Total CoQ10 ^a^ (oCoQ10) ^b^ (%)	k (Month^−1^)	R^2^	t_90%_ (Days)	t_90%_ (Months) ^c^
1	25.4 ± 0.4	99.8	81.1 (81.1)	0.149	0.943	21.2	9.7
2	4.2 ± 0.0	95.3	63.5 (62.1)	0.162	0.997	19.4	8.9
3	8.6 ± 0.2	99.4	63.3 (51.1)	0.227	0.968	13.9	6.3
4	25.2 ± 0.2	50.7	90.5 (67.5)	0.131	0.992	24.0	11.0
5	10.2 ± 0.3	34.0	103.2 (29.4)	0.392	0.940	8.0	3.7
8	46.1 ± 0.2	98.5	92.1 (91.1)	0.027	0.778	115.9	52.9
9	47.0 ± 1.2	91.3	94.5 (84.9)	0.143	0.989	22.1	10.1
10	22.0 ± 0.4	83.3	103.6 (61.4)	0.157	0.992	20.0	9.1
11	8.7 ± 0.2	27.8	95.0 (21.4)	1.346	1.000	2.3	1.1

^a^ Remaining total CoQ10 by the end of the accelerated stability study as a percentage of the initial total CoQ10; ^b^ Remaining oCoQ10 by the end of the accelerated stability study in relation to the initial oCoQ10 content (%)—most relevant in products with specified oCoQ10 content; ^c^ Predicted shelf-life by the Van’t Hoff equation.

**Table 3:** Stability of oCoQ10, expressed by first-order constant (day^−1^), in the presence of different concentrations of antioxidants and reducing agents.

Concentration	na	vit C	EA	vit E	Q	DTT	Na_2_S	FeCl_2_	βcar
High	S	0.048	S	S	S	1.822	1.489	S	S
Medium		0.022	S	S	S	0.025	0.350	S	S
Low		0.006	S	S	S	0.010	0.072	S	S

na—no addition; S—stable; vit C—vitamin C; EA—ellagic acid; vit E—vitamin E; Q—quercetin; DTT—1,4-dithiothreitol; Na_2_S—sodium sulfide; FeCl_2_—iron(II) chloride; βcar—β-carotene.

## Data Availability

Data is contained within the article or Supplementary Materials.

## Supplementary Materials

The following are available online at https://www.mdpi.com/2076-3921/10/3/360/s1.
