# Supplementary material for: Stability of Reduced and Oxidized Coenzyme Q10 in Finished Products
Source: Antioxidants (Basel). 2021 Feb 27;10(3):360. doi: 10.3390/antiox10030360 (PMC7997171; doi:10.3390/antiox10030360)
Supplement: Supplementary file 1 [file antioxidants-10-00360-s001.pdf]

Supplementary Materials

Journal name

Antioxidants

Article title

Stability of reduced and oxidized coenzyme Q10 in finished products

Authors

Žane Temova Rakuša, Albin Kristl, Robert Roškar*

University of Ljubljana, Faculty of Pharmacy, Aškerčeva cesta 7, 1000 Ljubljana, Slovenia

Tel: +386 1 4769 500, **e-mail:** robert.roskar@ffa.uni-lj.si

Brief description

Within Supplementary Material, we provide data on coenzyme Q10 (CoQ10) products' analysis from a technical point of view. It contains **three tables**, with information on a representative product containing mostly oxidized CoQ10 – oCoQ10 (**Table S1**), mostly reduced CoQ10 – rCoQ10 (**Table S2**), and a product containing significant parts of both oxidized and reduced CoQ10 (**Table S3**).

Representative chromatograms of these products at the beginning (time 0) and end (after 3 months) of the accelerated stability study (40°C, 75% RH) are provided, illustrating the difference in both sample preparation procedures (procedure I - extraction to determine the individual CoQ10 forms and procedure O - following oxidation with 0.01% Fe^{3+} solution in EtOH to determine total oCoQ10 content). The determined total CoQ10 content, expressed as a percentage of the label claim, by both sample preparation procedures (as a sum of the individual CoQ10 forms and total oCoQ10 content) are provided at both time points within the accelerated stability study along with the standard error of the mean (SEM), n=3. Retention times (tr) of the labeled chromatographic peaks are also provided.

Table S1 Representative chromatograms at the initial and last time point within the accelerated stability study of **product 3**, along with the determined total CoQ10 content, obtained by sample preparation I and O.

	Sample preparation I	Sample preparation O
Initial time point	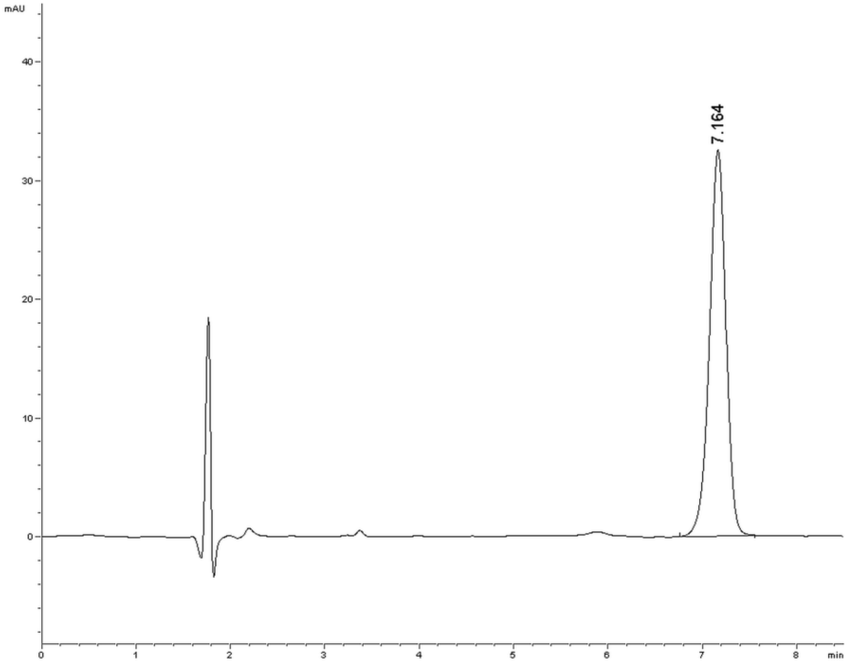	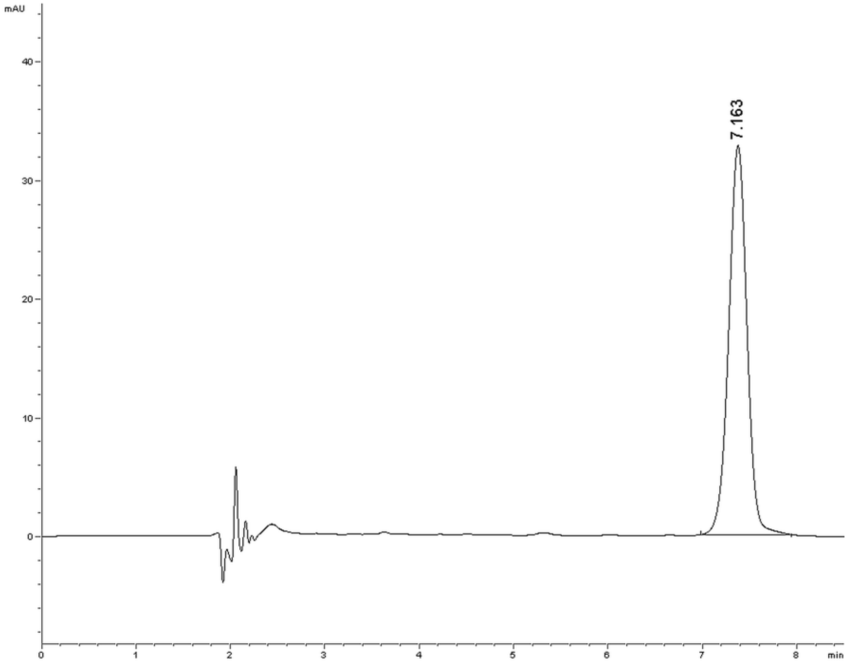
Labeled peak	oCoQ10 (tr 7.16)	oCoQ10 (tr 7.16)
Total CoQ10 content (% ± SEM)	86.6 ± 0.2	86.5 ± 0.1

	Sample preparation I	Sample preparation O
Last time point	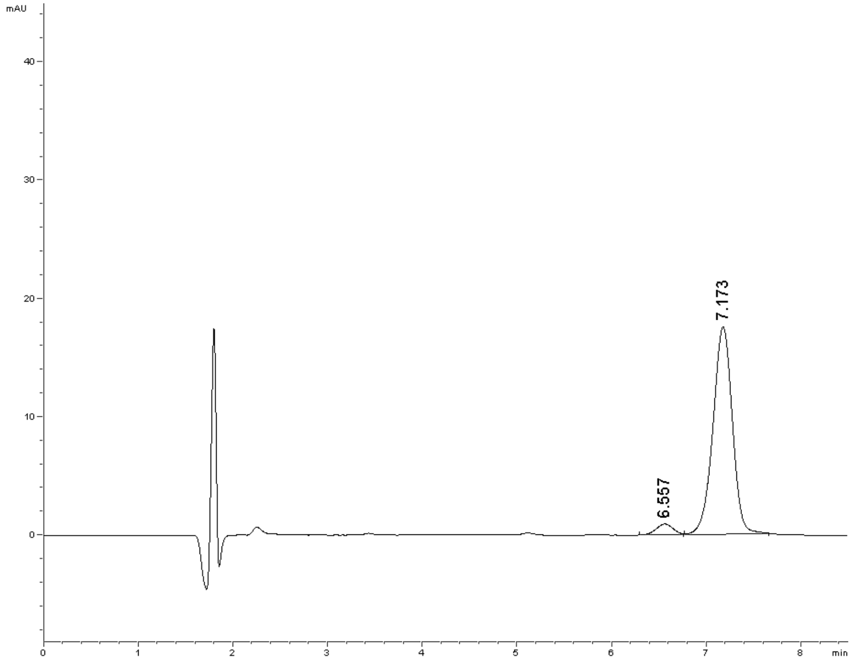	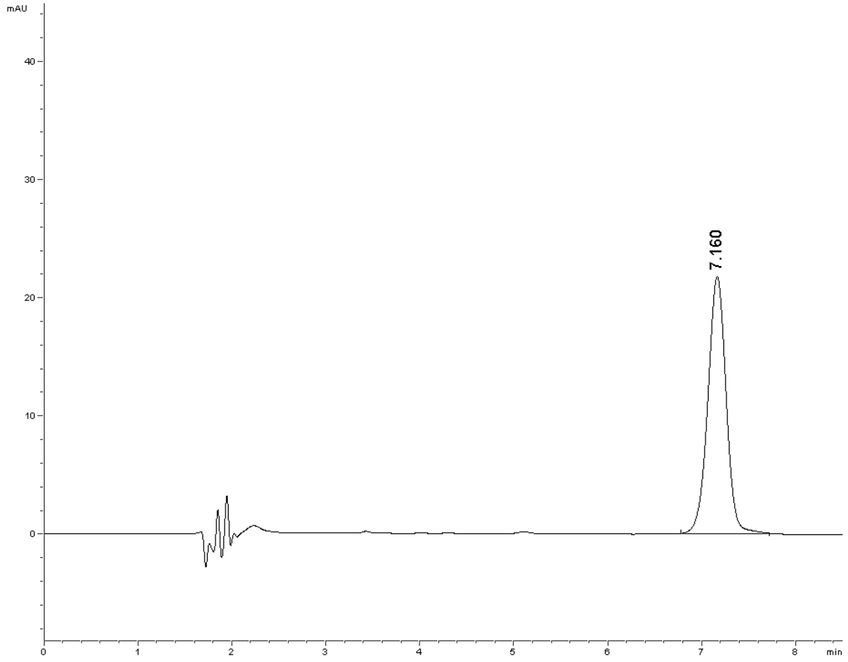
Labeled peaks	rCoQ10 (tr 6.56) & oCoQ10 (tr 7.17)	oCoQ10 (tr 7.16)
Total CoQ10 content (% ± SEM)	55.1 ± 0.3	58.2 ± 0.0

Table S2 Representative chromatograms at the initial and last time point within the accelerated stability study of **product 6**, along with the determined total CoQ10 content, obtained by sample preparation I and O.

	Sample preparation I	Sample preparation O
Initial time point	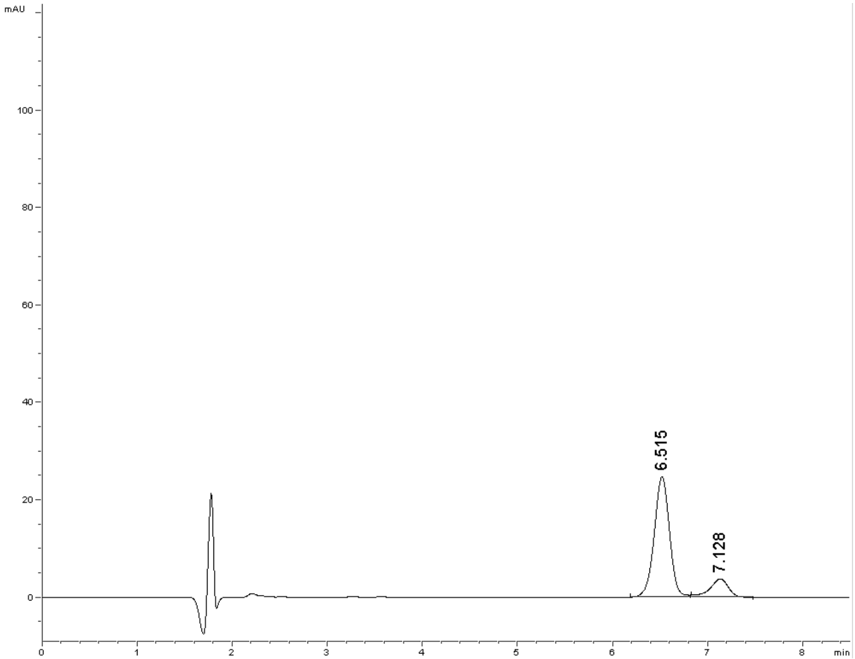	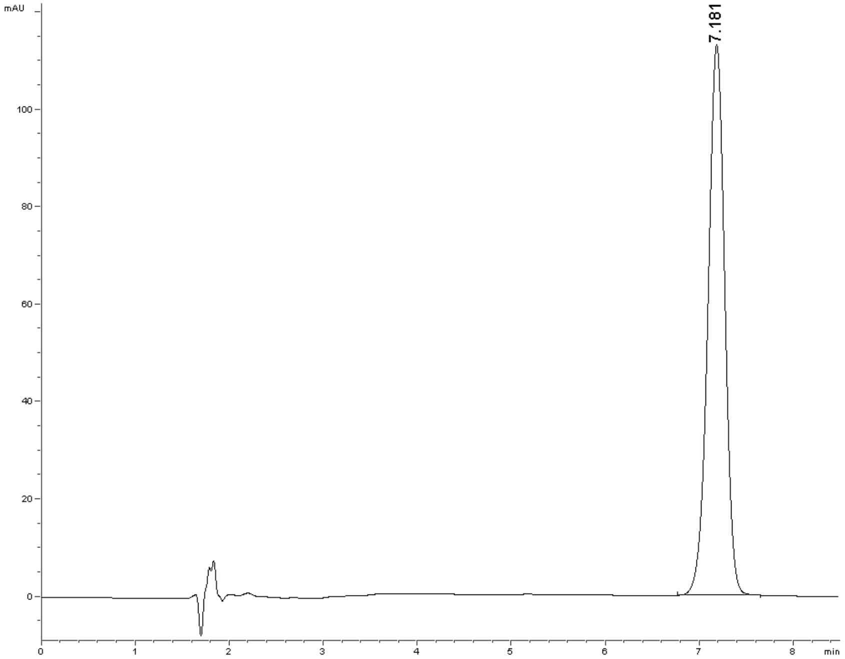
Labeled peaks	rCoQ10 (tr 6.51) & oCoQ10 (tr 7.13)	oCoQ10 (tr 7.18)
Total CoQ10 content (% ± SEM)	106.8 ± 0.1	104.9 ± 0.2

	Sample preparation I	Sample preparation O
Last time point	(chromatogram below)	(chromatogram below)
Labeled peaks	rCoQ10 (tr 6.63) & oCoQ10 (tr 7.26)	oCoQ10 (tr 7.26)
Total CoQ10 content (% ± SEM)	104.2 ± 0.8	102.1 ± 0.8

Sample preparation I

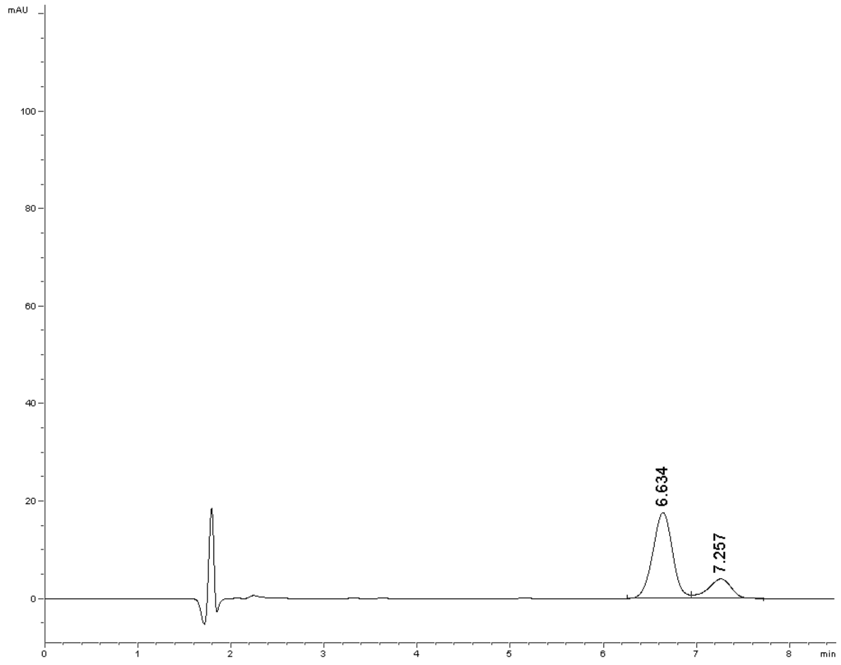

Sample preparation O

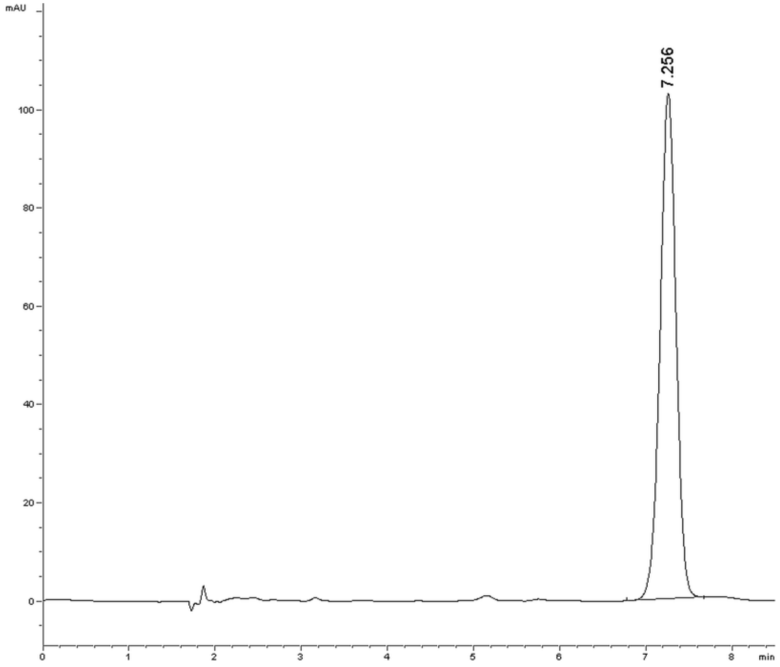

Table S3 Representative chromatograms at the initial and last time point within the accelerated stability study of **product 11**, along with the determined total CoQ10 content, obtained by sample preparation I and O.

	Sample preparation I	Sample preparation O
Initial time point	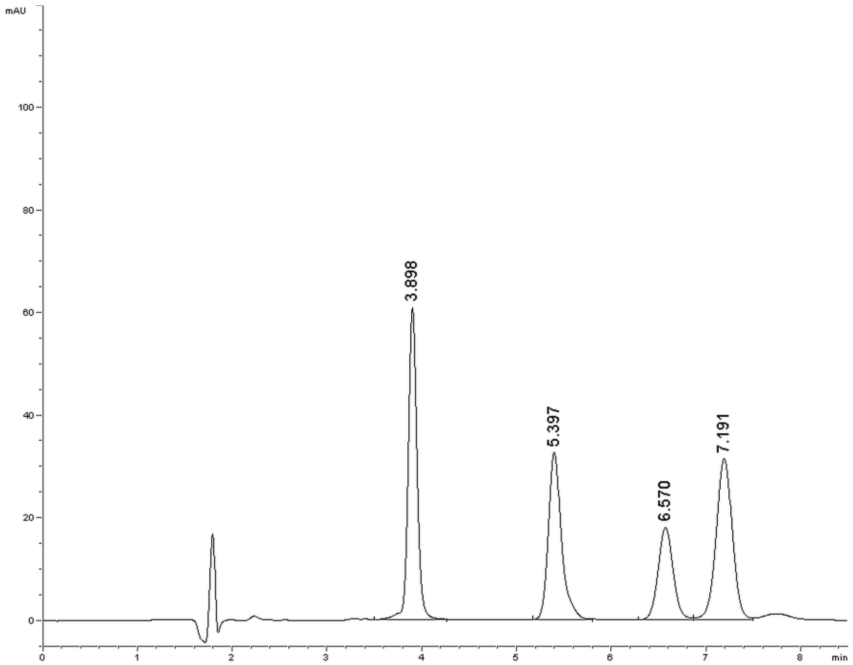	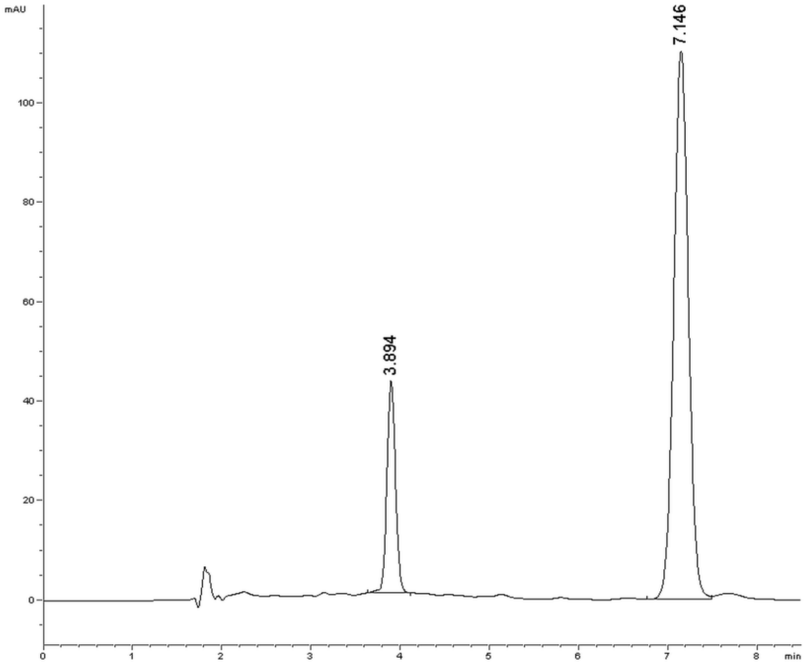
Labeled peaks	Tocoferyl acetate (tr 3.90), β-carotene (tr 5.40), rCoQ10 (tr 6.57) & oCoQ10 (tr 7.19)	Tocoferyl acetate (tr 3.89) & oCoQ10 (tr 7.15)
Total CoQ10 content (% ± SEM)	104.3 ± 0.2	103.6 ± 0.1

	Sample preparation I	Sample preparation O
Last time point	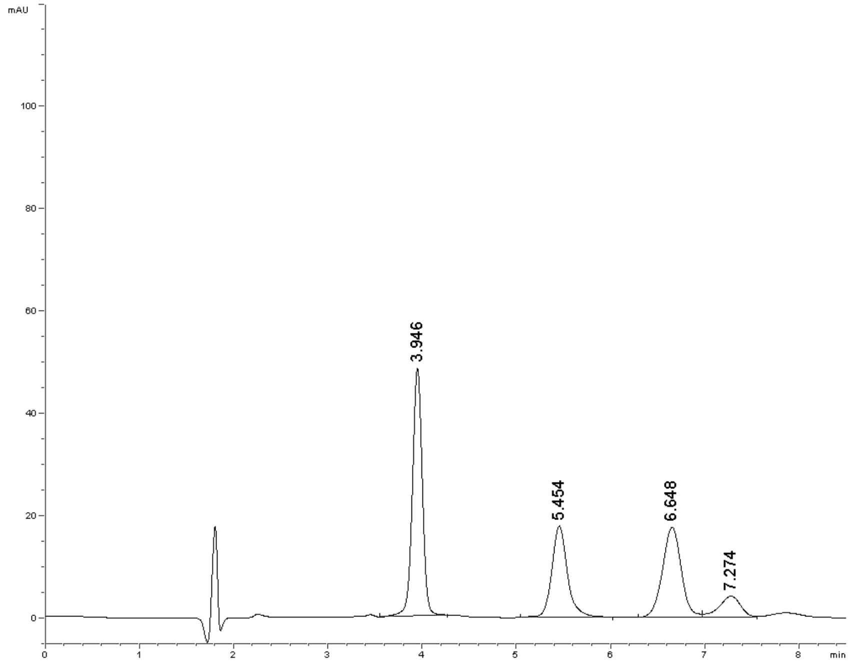	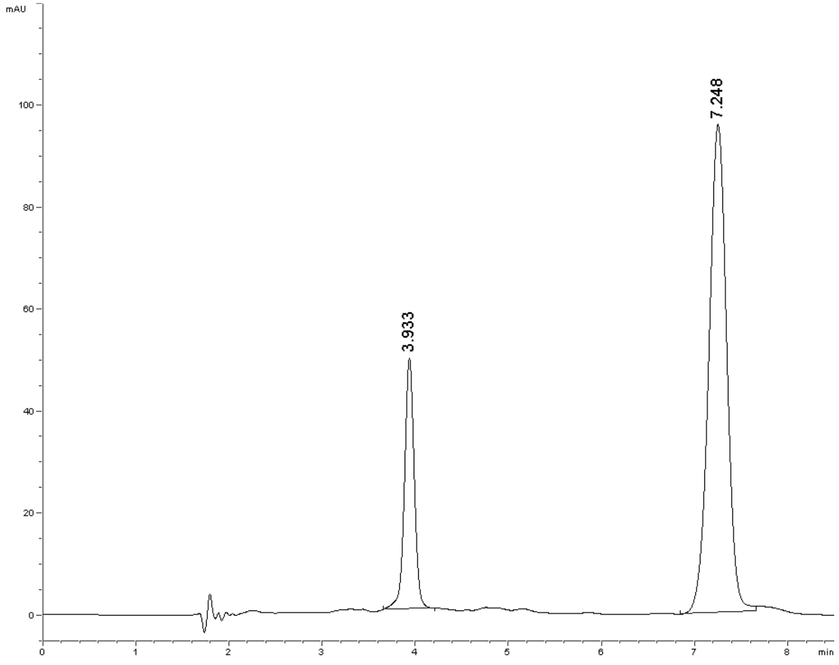
Labeled peaks	Tocoferyl acetate (tr 3.94), β-carotene (tr 5.45), rCoQ10 (tr 6.65) & oCoQ10 (tr 7.27)	Tocoferyl acetate (tr 3.93) & oCoQ10 (tr 7.25)
Total CoQ10 content (% ± SEM)	98.5 ± 0.4	98.5 ± 0.2
